# Comparison of resident COVID-19 mortality between unionized and nonunionized private nursing homes

**DOI:** 10.1371/journal.pone.0276301

**Published:** 2022-11-18

**Authors:** Adam Olson, Shivaram Rajgopal, Ge Bai

**Affiliations:** 1 Accounting Department, Lindner College of Business, University of Cincinnati, Cincinnati, Ohio, United State of America; 2 Accounting Division, Columbia Business School, Columbia University, New York, New York, United States of America; 3 Carey Business School and Bloomberg School of Public Health, Johns Hopkins University, Baltimore, Maryland, United States of America; Waikato Institute of Technology, NEW ZEALAND

## Abstract

Using bargaining agreement data from the Federal Mediation Conciliation Services, we found that the median national resident COVID-19 mortality percentage (as of April 24, 2022) of unionized nursing homes and that of nonunionized ones were not statically different (10.2% vs. 10.7%; *P* = 0.32). The median nursing home resident COVID-19 mortality percentage varied from 0% in Hawaii to above 16% in Rhode Island (16.6%). Unionized nursing homes had a statistically significant lower median mortality percentage than nonunionized nursing homes (*P* < 0.1) in Missouri, and had a higher median mortality percentage than nonunionized nursing homes (*P* < 0.05) in Alabama and Tennessee. Higher average resident age, lower percentage of Medicare residents, small size, for-profit ownership, and chain organization affiliation were associated with higher resident COVID-19 mortality percentage. Overall, no evidence was found that nursing home resident COVID-19 mortality percentage differed between unionized nursing homes and nonunionized nursing homes in the U.S.

## Introduction

As of April 24, 2022, 152,324 nursing home residents in the U.S. had died from COVID-19, representing 15.4% of all COVID-19 deaths (990,769) in the U.S. [1, 2]. Understanding the factors associated with resident mortality has important implications for infection control policies and practice [3]. Two recent study found a negative association between nursing home unionization and resident COVID-19 mortality percentage [4, 5]. However, the unionization measure in these studies was obtained from a proprietary source [6, 7], and these studies’ categorization of all nursing homes with one or more union-affiliated employees as unionized nursing homes regardless of any actual bargaining agreements or contracts. For example, a nursing home that employs a union-affiliated non-nursing staff such as a janitor but has no actual bargaining contract with any union is considered unionized nursing homes in these studies. In this study, we improved the measurement of unionization by using publicly available bargaining agreement data and defining a nursing homes as unionized if it has a contract with one or more unions.

Prior research gives little insight into how unionization will affect nursing home COVID-19 mortality. Unionization has been found to lead to no differences in nursing home care quality, increased employee pay, and decreased employee numbers [8]. Mandatory overtime restrictions, popular among unions, have been shown to lower care quality [9]. Yet unionized nursing homes were more likely to have appropriate protective equipment in the early stages of the COVID-19 pandemic [5]. We aim to understand the association between nursing home unionization and resident COVID-19 mortality percentage by examining publicly available unionization information and national nursing home resident COVID-19 mortality data as of April 24, 2022, which captures most nursing home resident COVID-19 mortality in light of the mortality trajectories [2].

## Materials and methods

### Data and sample

Nursing home resident COVID-19 mortality percentages (as of April 24, 2022) were downloaded from the Centers for Medicare and Medicaid Services (CMS) COVID-19 Nursing Home Data website on May 12, 2022 (a data source separate from hospitals) [1]. A total of 15,486 nursing homes were in the dataset. After excluding 1,106 government nursing homes (approximately 7%), whose unionization information is not publicly available, the full dataset included 14,380 nursing homes. We examine these nursing homes in a retrospective cohort study where unionization determines cohort status.

The CMS dataset also contained nursing home size (the average occupied beds). From CMS’ Provider Information website, we obtained nursing home ownership type and five-star quality rating [10]. From Long-Term Care Focus at Brown University School of Public Health, we gathered the average age of residents, chain organization affiliation, percent of residents enrolled in the Medicare program, percent of residents enrolled in the Medicaid program, percent of obese residents, registered nurse (RN) hours per patient, licensed practical nurse (LPN) hours per patient, certified nursing assistant (CNA) hours per patient, resident acuity, and percent of white residents [11]. These three datasets were merged by using nursing homes’ federal provider identification numbers.

Moreover, we calculated county-level COVID-19 infection percentages by using information downloaded from USAFacts on May 12, 2022 [12], and merged them with the nursing home dataset based on Federal Information Processing Standards (FIPS) county codes. All variables obtained were consistent with those examined in prior research [4, 5]. The merged dataset included 7,509 nursing homes with non-missing values on all variables. See Table 1 for further details regarding our sample and sources.

**Table 1:** Data sources and variables.

Data Set	Source	When Accessed	Variable Used to Merge
**COVID-19 Nursing Home Data**	https://data.cms.gov/stories/s/bkwz-xpvg	5/12/22	
	Variables: Name, Federal provider number, COVID-19 deaths, Number of occupied beds		
**Union F-7 Filings**	https://www.fmcs.gov/resources/documents-and-data/	5/12/22	Name
	Variables: Name, F-7 contract (unionized)		
**Center for Medicare and Medicaid Services Nursing Home Provider Information**	https://data.cms.gov/provider-data/dataset/4pq5-n9py	11/20/20	Federal Provider Number
	Variables: County SSA code, For-profit ownership, Overall quality rating (5-star system)		
**Brown University Nursing Home Data**	http://ltcfocus.org/	11/20/20	Federal Provider Number
	Variables: % Medicare residents, % Medicaid residents, % white residents, % obese residents, RN-to-bed ratio, LPN-to-bed ratio, CAN-to-bed ratio, Resident acuity, Average age of residents, Chain organization affiliation, Occupancy %		
**County COVID-19 Data**	https://usafacts.org/visualizations/coronavirus-covid-19-spread-map/	5/12/22	County FIPS Code
	Variables: County population, County COVID-19 infection percentage, County FIPS		
**County FIPS to SSA Code**	https://www.nber.org/research/data/ssa-federal-information-processing-series-fips-state-and-county-crosswalk	11/11/20	County SSA Code
	Variables: County FIPS, County SSA code		

**Notes:** Before names were used to merge Union F-7 Filings, names were changed to all lower-case and non-alphanumeric characters were removed. Stata’s reclink command was used to obtain potential name matches. All potential matches were examined by hand at least twice to make sure they were the same nursing home. If the entire nursing home chain had an F-7 filing associated with it, we marked each individual nursing home as unionized. If we were not certain of the name match, we erred on not matching them as to not add bias to our sample by listing nonunionized nursing homes as unionized. This process is not perfect and is a limitation of the study. However, the differences between unionized and nonunionized nursing homes shown in Table 3 suggest that this process did split our sample into two distinct groups. Abbreviations: FIPS: Federal Information Processing Standards; SSA: Social Security Administration; RN: registered nurse; LPN: licensed practical nurse; CNA: certified nursing assistant.

### Measurement of unionization

We define a nursing home as unionized if that nursing home has an active contract with at least one union. Consistent with prior literature, we measure nursing home unionization status using data from the Federal Mediation Conciliation Services (FMCS) [6]. In the U.S., a Notice of Bargaining (F-7 Notice) must be filed with FMCS within 30 days of the beginning of negotiations for a new or modified collective bargaining agreement between an employer and a union [13]. F-7 Notices filed are published on the website of FMCS [14]. Among the published F-7 Notices, using nursing home names, we identified 1,293 nursing homes that filed F-7 Notices between January 1, 2015 and December 31, 2019—approximately five years before the measurement of COVID-19 mortality percentages until shortly before the COVID-19 pandemic—to be consistent with the methodology used in prior research [6]. We considered these 1,293 nursing homes as unionized and the remaining 13,087 nursing homes as nonunionized. The proportion of unionized nursing homes (9.0%) in the sample is consistent with the results in prior research and national statistics on unionization in the private sector [6, 7].

### Statistical analysis

Using the full dataset that contained 14,380 nursing homes, we compared the median resident COVID-19 mortality percentage between unionized and nonunionized nursing homes nationwide. Throughout this study, all tests of medians are a nonparametric k-sample test on the equality of medians with continuity correction. We also conducted the same analysis for each state and the District of Columbia, respectively. We calculate nursing homes COVID-19 mortality percentage as the number of deaths of residents of nursing homes attributed to COVID-19 divided by the average number of occupied beds during our sample period. CMS includes all COVID-19 deaths of nursing home patients regardless of where the actual death occurs.

Using the merged dataset that contained 7,509 nursing homes, consistent with prior literature [4, 5], we compared county-level population and COVID-19 infection percentage, average age of residents, percent of residents enrolled in the Medicare program, percent of residents enrolled in the Medicaid program, percent white residents, RN hours per patient, LPN hours per patient, CNA hours per patient, five-star quality rating, percent obese residents, resident acuity, size, ownership type, chain organization affiliation, and occupancy percentage for unionized nursing homes and nonunionized ones.

We also conducted multivariate regression analysis using the ordinary least squares (OLS) method to examine the association between nursing home unionization as well as aforementioned nursing home and locality characteristics, and the resident COVID-19 mortality percentage. County fixed effects were added to control for unobserved factors that vary across counties. Standard errors were clustered at the county level.

## Results

### National level comparison (full dataset)

**Fig 1:**
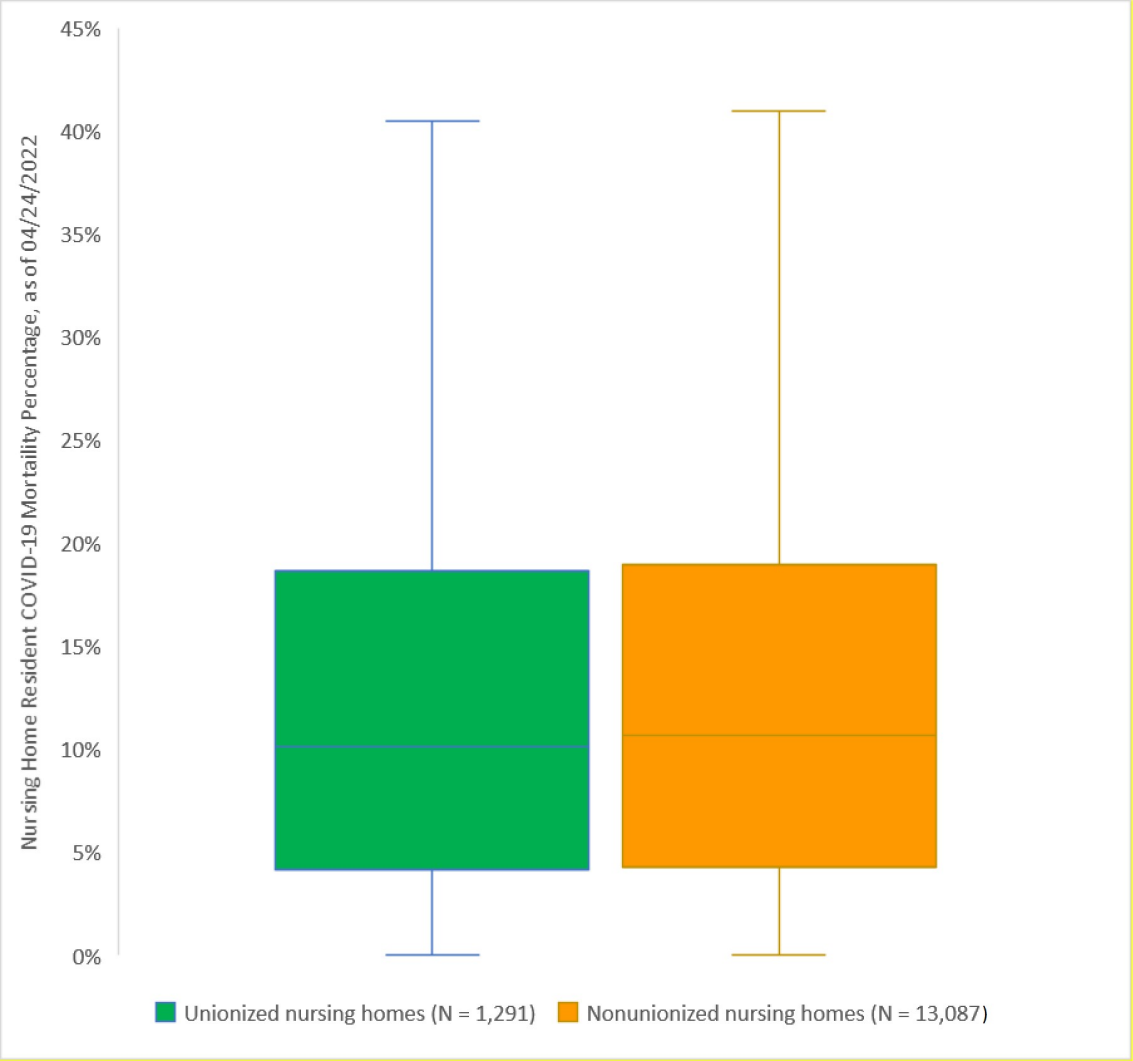
Nationwide private nursing home resident mortality percentages among unionized (N = 1,291) and nonunionized (N = 13,087) nursing homes, as of April 24, 2022. **Note:** The full dataset that contained 14,380 nursing homes was used. Box plot lines represent the 25th percentile, median, and 75th percentile. Whiskers are 1.5 times the interquartile ranges. The median COVID-19 mortality percentage for unionized nursing homes is not statistically significantly differently from that for nonunionized nursing homes (10.15% vs. 10.68%; *P* = 0.32) when using a nonparametric k-sample test on the equality of medians with continuity correction.

As of April 24, 2022, the median mortality percentage of 1,293 unionized nursing homes is 10.2%, and the median mortality percentage of 13,087 nonunionized nursing homes is 10.7%. The two median values are not statically different (*P* = 0.32) (Fig 1).

### State-level comparison (full dataset)

As shown in Table 2, the proportion of unionized nursing homes differed across states, varying from 0% in Wyoming to above 20% in Michigan (25.7%), Hawaii (23.7%), New Jersey (22.4%), Minnesota (20.1%), and New York (20.1%). The median nursing home resident COVID-19 mortality percentage, as of April 24, 2022, varied from 0% in Hawaii to above 15% in Rhode Island (16.6%), Connecticut (16.0%), and Indiana (15.3%). Unionized nursing homes had a statistically lower median mortality percentage than nonunionized nursing homes (*P* < 0.1) in one state (Missouri), and had a statistically higher median mortality percentage than nonunionized nursing homes (*P* < 0.05) in two states (Alabama and Tennessee).

**Table 2:** Private nursing home resident COVID-19 mortality percentages, by state.

State	# of private nursing homes	# of unionized	# of nonunionized	% unionized	Median mortality % (all)	Median mortality % (unionized)	Median mortality % (nonunionized)
Alaska	14	1	13	7.14%	1.36%	0.00%	1.63%
Alabama	214	17	197	7.94%	11.11%	21.26%***	10.68%***
Arkansas	213	2	211	0.94%	13.71%	8.12%	13.82%
Arizona	142	7	135	4.93%	9.64%	5.23%	9.75%
California	1,135	152	983	13.39%	8.49%	8.62%	8.47%
Colorado	208	6	202	2.88%	11.60%	12.09%	11.60%
Connecticut	210	27	183	12.86%	15.97%	18.98%	15.62%
Washington DC	19	2	17	10.53%	6.99%	3.42%	7.36%
Delaware	45	6	39	13.33%	11.30%	13.29%	10.72%
Florida	688	84	604	12.21%	6.54%	6.80%	6.48%
Georgia	343	3	340	0.87%	10.32%	7.04%	10.43%
Hawaii	38	9	29	23.68%	0.00%	1.13%	0.00%
Iowa	426	25	401	5.87%	11.08%	7.88%	11.12%
Idaho	71	4	67	5.63%	8.11%	9.74%	7.89%
Illinois	693	110	583	15.87%	11.71%	9.98%	12.22%
Indiana	395	8	387	2.03%	15.31%	17.75%	15.18%
Kansas	292	29	263	9.93%	10.99%	15.28%	10.84%
Kentucky	277	7	270	2.53%	11.36%	9.93%	11.44%
Louisiana	263	2	261	0.76%	10.68%	20.13%	10.62%
Massachusetts	371	14	357	3.77%	14.38%	11.02%	14.47%
Maryland	220	20	200	9.09%	9.45%	11.70%	9.32%
Maine	92	4	88	4.35%	9.16%	10.48%	9.16%
Michigan	401	103	298	25.69%	11.46%	11.32%	11.54%
Minnesota	334	67	267	20.06%	9.97%	9.59%	10.00%
Missouri	491	25	466	5.09%	10.14%	7.68%*	10.42%*
Mississippi	178	15	163	8.43%	12.39%	19.20%	12.28%
Montana	62	4	58	6.45%	13.91%	18.77%	13.44%
North Carolina	413	26	387	6.30%	11.47%	12.53%	11.28%
North Dakota	77	1	76	1.30%	12.53%	4.95%	12.62%
Nebraska	162	3	159	1.85%	8.39%	22.25%	8.29%
New Hampshire	64	1	63	1.56%	10.24%	9.91%	10.57%
New Jersey	349	78	271	22.35%	13.72%	12.77%	13.76%
New Mexico	67	1	66	1.49%	9.17%	17.50%	8.99%
Nevada	59	5	54	8.47%	8.71%	0.00%	8.95%
New York	582	117	465	20.10%	8.10%	7.65%	8.32%
Ohio	945	77	868	8.15%	12.23%	12.04%	12.37%
Oklahoma	283	5	278	1.77%	11.83%	7.39%	12.07%
Oregon	127	1	126	0.79%	5.37%	2.42%	5.46%
Pennsylvania	655	119	536	18.17%	14.58%	13.00%	14.70%
Rhode Island	80	3	77	3.75%	16.58%	14.31%	16.72%
South Carolina	176	2	174	1.14%	12.47%	7.34%	12.47%
South Dakota	99	4	95	4.04%	13.77%	11.47%	13.77%
Tennessee	301	6	295	1.99%	12.89%	17.38%**	12.35%**
Texas	1,077	16	1,061	1.49%	9.94%	11.53%	9.94%
Utah	72	4	68	5.56%	5.11%	4.38%	5.15%
Virginia	281	11	270	3.91%	10.54%	10.95%	10.47%
Vermont	34	1	33	2.94%	4.14%	2.74%	4.50%
Washington	193	15	178	7.77%	7.97%	8.39%	7.92%
Wisconsin	306	34	272	11.11%	9.33%	11.03%	9.08%
West Virginia	121	10	111	8.26%	10.67%	14.20%	10.50%
Wyoming	22	0	22	0.00%	11.81%	Not applicable	11.81%

**Note:** The full dataset that contained 14,380 nursing homes was used.

*P < 0.10

**; *P* < 0.05

***P < 0.01 for two-tailed nonparametric k-sample test on the equality of medians with continuity correction between unionized and nonunionized nursing homes. Orange shade indicates higher mortality in unionized nursing homes.

### Nursing home characteristics (merged dataset)

Table 3 shows the comparison results among the 7,509 nursing homes with non-missing values on all variables extracted. Compared to residents in nonunionized nursing homes, residents in unionized nursing homes were younger (median: 78.13 vs. 79.85; P < 0.01), in larger counties (median: 522,798 vs. 305,506; P < 0.01) with lower COVID-19 infection percentages (median: 23.82 vs. 24.09%; P < 0.05), and less likely to be white (median: 79.34% vs. 86.08%; P < 0.01). Unionized nursing homes were larger (median: 91.46 vs. 81.46 average occupied beds; P < 0.01), had higher occupancy percentages (median: 87.50% vs. 86.54%; P < 0.01), and had a higher proportion of Medicaid residents (median: 67.17% vs. 65.00%, P < 0.05), all consistent with Sojourner et al. (2010). Further, unionized nursing homes had a relatively high RN-to-bed ratio (median: 0.44 vs. 0.41; P < 0.1), relatively low LPN-to-bed ratio (median: 0.82 vs. 0.85; P < 0.01) and CNA-to-bed ratio (median: 2.19 vs. 2.25; P < 0.1).

**Table 3:** Comparison of nursing home characteristics, by unionization.

Variable	Unionized Nursing Homes				Nonunionized Nursing Homes				Differences in Medians
	(N = 866)				(N = 6,643)				
	25^th^ Percentile	Median	75^th^ Percentile	Standard Deviation	25^th^ Percentile	Median	75^th^ Percentile	Standard Deviation	
County population	150,500	522,798	1,200,000	1,600,000	75,783	305,506	854,757	1,800,000	217,292***
County COVID-19 infection %	21.41%	23.82%	26.58%	4.12%	21.62%	24.09%	27.33%	4.31%	-0.27%**
Average age of residents	74.00	78.13	82.44	6.13	75.63	79.85	83.37	5.98	-1.72***
% Medicare residents	6.42%	11.36%	17.39%	10.14%	6.58%	11.25%	17.46%	10.60%	0.11%
% Medicaid residents	54.55%	67.17%	77.55%	18.83%	52.38%	65.00%	75.68%	19.34%	2.17%**
% white residents	53.75%	79.34%	93.27%	24.58%	65.91%	86.08%	95.95%	22.18%	-6.74%***
Registered-nurse-to-bed ratio	0.29	0.44	0.63	0.27	0.27	0.41	0.59	0.28	0.03*
Licensed-practical-nurse-to-bed ratio	0.65	0.82	0.98	0.30	0.67	0.85	1.03	0.31	-0.03***
Certified-nursing-assistant-to-bed ratio	1.94	2.19	2.52	0.51	1.96	2.25	2.60	0.54	-0.06***
5-star quality rating	2.00	3.00	4.00	1.39	2.00	3.00	4.00	1.40	-0.00**
% obese residents	22.52%	27.11%	32.26%	7.60%	22.54%	27.40%	32.50%	7.61%	-0.29%
Resident acuity	1.17	1.23	1.29	0.11	1.18	1.24	1.29	0.11	-0.01
# of occupied beds	70.58	91.46	120.74	44.94	61.14	81.46	104.91	39.25	10.00***
For-profit ownership	0.00	1.00	1.00	0.46	1.00	1.00	1.00	0.41	0.00
Chain organization affiliation	0.00	1.00	1.00	0.49	0.00	1.00	1.00	0.48	0.00
Occupancy %	79.66%	87.50%	93.18%	11.41%	76.53%	86.54%	92.59%	12.85%	0.96%***

**Note:** The merged dataset that contained 7,509 nursing homes was used.

*P < 0.10

**; *P* < 0.05

***P < 0.01 for two-tailed nonparametric k-sample test on the equality of medians with continuity correction between unionized and nonunionized nursing homes. County COVID-19 infection percentage is as of April 24, 2022.

### Regression results (merged dataset)

As presented in Table 4, unionization was not statistically significantly associated with the nursing home COVID-19 mortality percentage (coefficient = -0.0018; P = 0.75). Other things equal, a one-year increase in the average age of residents was associated with 0.3% higher COVID-19 mortality percentage (P < 0.01); having 10 more occupied beds was associated with 0.1% lower mortality percentage (P < 0.1); for-profit ownership was associated with 1.3% higher mortality percentage (P < 0.05), and chain organization affiliation was associated with 0.7% higher mortality percentage (P < 0.05). In addition, percent of residents enrolled in the Medicare program had a small association with mortality percentage—a 1% higher enrollment in Medicaid program was associated with 0.06% lower mortality percentage (P < 0.01).

**Table 4:** Factors associated with nursing home resident COVID-19 mortality percentage, as of April 24, 2022.

Variable	Estimated Coefficient
Unionization	-0.0018
Average age of residents	0.0033***
% Medicare residents	-0.0579***
% Medicaid residents	-0.0028
% white residents	0.0361***
Registered-nurse-to-bed ratio	0.0032
Licensed-practical-nurse-to-bed ratio	-0.0088
Certified-nursing-assistant-to-bed ratio	-0.0049
2-star quality rating	0.0067
3-star quality rating	0.0024
4-star quality rating	-0.0002
5-star quality rating	-0.0069
Obese percentage	-0.0183
Resident acuity	0.0011
# of occupied beds	-0.0001*
For-profit ownership	0.0125**
Chain organization affiliation	0.0073**
Occupancy %	-0.0081
County fixed effects	Yes
Adjusted R^2^	0.097
N	7,509

**Note:** The merged dataset that contained 7,509 nursing homes was used. *P < 0.10 **; *P* < 0.05; ***P < 0.01. Ordinary Least Squares method was used, with standard errors clustered at the county level.

We conducted two sensitivity tests to understand whether our results are sensitive to excluded nursing homes due to their missing value on at least one control variables in the regression. First, we dropped all control variables obtained from the Brown University data from our regression. Second, we set all missing values of control variables from the Brown University data to the remaining observations’ mean values. In both cases, the association between unionization and nursing home COVID-19 mortality remained statistically insignificant (p-values of 0.49 and 0.83, respectively).

## Discussion

Based on COVID-19 mortality data as of April 22, 2022, unionized nursing homes did not have significantly different COVID-19 mortality from nonunionized nursing homes. We also found robust evidence that higher average resident age, lower percentage of Medicare residents, small size, for-profit ownership, and chain organization affiliation were associated with a higher resident COVID-19 mortality percentage, consistent with prior literature on risk factors affecting nursing home COVID-19 mortality [15, 16]. Compared to the prior research that examined nursing home unionization and resident COVID-19 mortality [4, 5], our measurement of unionization has two main strengths [6, 7]. First, our definition of unionization is not based on the presence of employees who are members of a union, but based on the existence of a union contract. Without a contract in place, it is unclear that a union member employee could have any bargaining power beyond that of other employees (i.e., the ability to leave). However, once the contract is in place, the nursing home is required to meet certain demands of employees. Therefore, focusing on contracts allows us to measure when the union has real power within the nursing home to impact policies and make changes. Second, instead of a proprietary source, we used publicly available bargaining agreement data from FMCS and methodology adopted in prior literature to identify unionized nursing homes, making our analysis replicable [6].

The validity of our results is subject to measurement and reporting noises in COVID-19 mortality data. Moreover, because nursing homes are not required to file the F7 Notice if they do not sign an agreement with a labor union, nursing homes that hired workers from labor unions but did not sign contracts with the union were identified as nonunionized in this study. Furthermore, nursing homes with F7 Notices must be matched by name to nursing home COVID-19 data, creating some noise in the union measure. Also, the variation of resident COVID-19 mortality percentages across nursing homes is partially attributable to the variation of residents’ underlying medical conditions [17], for which the national data is unavailable. Moreover, we do not have access to data on care quality, which prevented us from understanding the care difference between unionized and nonunionized nursing homes. In addition, the trend of unionization in nursing homes is beyond the scope of this study. Finally, our regression analysis was conducted on a subsample due to the requirement of non-missing value for all control variables, and thus the results might be subject to selection biases. In sensitivity tests that dropped all control variables and imputed missing data as the mean of the non-missing sample, our results remain qualitatively unchanged.

Understanding risk factors affecting nursing home COVID-19 mortality is critical to inform policymakers, community leaders, and nursing home stakeholders aiming at improving infection control in nursing homes [3]. Numerous research has established the association between key nursing home characteristics—such as location, size, and staffing levels—and resident COVID-19 mortality [18]. Two studies to date, using proprietary unionization data inconsistent with prior research, has found an association between nursing home unionization and resident COVID-19 mortality [4, 5]. The results of this study, derived from national mortality and publicly available unionization data, provides no evidence to support a relationship between nursing home unionization and resident COVID-19 mortality.

## Conclusions

As of April 24, 2022, Nursing home residents accounted for 15.4% of deaths from COVID-19 in the U.S. Using national COVID-19 mortality data as of April 24, 2022 and publicly available bargaining agreement data from FMCS, we found no evidence that nursing home resident COVID-19 mortality percentage differed between unionized nursing homes and nonunionized nursing homes. Higher average resident age, lower percentage of Medicare residents, small size, for-profit ownership, and chain organization affiliation were associated with higher resident COVID-19 mortality percentage. These findings contribute to the literature on risk factors affecting nursing home COVID-19 mortality and inform public policies aimed at infection control in nursing homes [16, 18].
